# Fecal carriage of multidrug-resistant *Escherichia coli* by community children in southern Taiwan

**DOI:** 10.1186/s12876-018-0807-x

**Published:** 2018-06-15

**Authors:** I-Fei Huang, Wei-Yang Lee, Jiun-Ling Wang, Chih-Hsin Hung, Hong-Hsiang Hu, Wan-Yu Hung, Yun-Ju Hung, Wen-Chi Chen, Ying-Tso Shen, Ming-Fang Cheng

**Affiliations:** 1 Department of Pediatrics, Kaohsiung Veterans General Hospital, No. 386, Ta-Chung 1st Road, Kaohsiung, 81362 Taiwan; 2 School of Medicine, National Yang-Ming University, Taipei, Taiwan; 3 Chung Hwa University of Medical Technology, Tainan, Taiwan; 4 Department of Pediatrics, Kaohsiung Municipal United Hospital, Kaohsiung, Taiwan; 5 Department of Internal Medicine, College of Medicine, National Cheng Kung University Hospital, National Cheng Kung University, Tainan, Taiwan; 6 Department of Medicine, College of Medicine, National Cheng Kung University, Tainan, Taiwan; 7 Department of Chemical Engineering and Institute of Biotechnology and Chemical Engineering, I-Shou University, Kaohsiung, Taiwan; 8 Division of Gastroenterology, Department of Medicine, Kaohsiung Veterans General Hospital, Kaohsiung, Taiwan; 9 Fooyin University, Kaohsiung, Taiwan

**Keywords:** *Escherichia coli*, Fecal carriage, Multidrug-resistant, Extended-spectrum β-lactamase, Children

## Abstract

**Background:**

The emergence of multidrug-resistant (MDR) *Escherichia coli* (*E. coli*), particularly *E. coli* sequence type ST131, is becoming a global concern. Commensal bacteria, an important reservoir of antibiotic resistance genes, facilitate the spread of such genes to pathogenic bacterial strains. The objective of the study is to investigate the fecal carriage of MDR *E. coli* and ST131 *E. coli* in community children in Southern Taiwan.

**Methods:**

In this prospective study, stool samples from children aged 0–18 years were obtained within 3 days of hospitalization from October 2013 to September 2014. Children with a history of underlying diseases, antibiotic treatment, or hospitalization in the 3 months before specimen collection were excluded. *E. coli* colonies were selected and tested for antimicrobial susceptibility, and O25b-ST131, multilocus sequence typing, and blaCTX-M gene groups were detected.

**Results:**

Among 157 *E. coli* isolates, the rates of nonsusceptibility to ampicillin, amoxycillin + clavulanate, trimethoprim–sulfamethoxazole, and cefazolin were 70, 65.6, 47.1, and 32.5%, respectively. Twenty-nine (18.5%) isolates were nonsusceptible to ciprofloxacin. MDR *E. coli* accounted for 58 (37%) of all isolates. Thirteen (8.3%) isolates produced extended-spectrum β-lactamase (ESBL). Furthermore, 26 (16.6%) and 13 (8.3%) isolates were O25b and ST131 positive, respectively. Five (38.5%) of the 13 ESBL-producing *E. coli* belonged to blaCTX-M group 9, among which were CTXM-14 and 4 (80%) were O25b–ST131 positive. Compared with the non-ESBL and ciprofloxacin-susceptible groups, the ESBL and ciprofloxacin-nonsusceptible groups showed significantly higher rates of O25b–ST131 positivity.

**Conclusions:**

The prevalence of the fecal carriage of nonsusceptible *E. coli* in children was high; among these *E. coli*, 37% were MDR, 18.5% were nonsusceptible to ciprofloxacin, and 8.3% produced ESBL. O25b–ST131 was the most common ESBL-producing *E. coli* clonal group present in the feces of children, and the ESBL and ciprofloxacin-nonsusceptible groups showed significantly higher rates of O25b–ST131 positivity.

**Electronic supplementary material:**

The online version of this article (10.1186/s12876-018-0807-x) contains supplementary material, which is available to authorized users.

## Background

The species of the *Escherichia* genus is heterogeneous, and this genus includes both commensal and pathogenic bacteria. Although only some *E. coli* are pathological species, they cause infections in various organs, such as the urinary tract, biliary system, and central nervous system, ranging from spontaneously resolving cystitis to life-threatening sepsis syndrome in humans of all ages [1]. Increasing antibiotic resistance results in increased mortality and morbidity, enhances transmission of resistant bacteria, and increases health expenses [2]. The emergence of MDR *E. coli* is becoming a global concern, with particular emphasis on *E. coli* sequence type ST131, which is increasingly reported in urinary tract infections (UTIs). In 2008, *E. coli* ST131 was identified as a major clone associated with the spread of CTX-M-15 ESBL resistance [3–5]. Thereafter, *E. coli* ST131 was also strongly associated with fluoroquinolone resistance and co-resistance to aminoglycosides and trimethoprim–sulfamethoxazole (TMP–SMZ) [6–8]. Current strategies to monitor antibiotic resistance in bacteria mainly rely on examining resistance in pathogenic organisms [9]. However, commensal bacteria, an important reservoir of antibiotic resistance genes, facilitate the spread of such genes to pathogenic bacterial strains [10, 11]. Humans, companion and noncompanion animals, and foods are established reservoirs of the ST131 *E. coli* clone [12]. Few studies have investigated fecal carriage of MDR *E. coli* by community children. Therefore, the objective of the study was to investigate the fecal carriage of MDR *E. coli* and ST131 *E. coli* by community children in Southern Taiwan.

## Methods

### Study population

In this prospective study, children aged 0–18 years who were admitted to the Pediatric Department of Kaohsiung Veterans General Hospital from October 2013 to September 2014 because of mild febrile illnesses, namely acute respiratory, gastroenteritis, or skin and soft tissue infection, and underwent regular examinations were enrolled. Exclusion criteria were any history of antibiotic treatment or hospitalization in the 3 months before specimen collection and a history of underlying diseases. The study was approved by the Ethics Committee of the Kaohsiung Veterans General Hospital (reference number VGHKS16-CT2–04). All participants (their parent or legal guardian in the case of children aged less than 16 years) provided informed consent. Stool samples were obtained as soon as it was available after admission. All of them were obtained within 3 days of admission [13].

### Microbiological laboratory procedures

#### Screening for *E. coli* strains in stool samples

Each stool sample was spread on a CHROMagar™ ECC plate (CHROMagar, Paris, France), which is efficient for the simultaneous enumeration of *E. coli* [14] and incubated at 37 °C for 24 h; *E. coli* strains appeared as blue colonies. A blue colony was picked [14] and added to 1 mL of sterilized Luria–Bertani medium for serial dilution. A 100-μL suspension was subsequently spread on the CHROMagar™ ECC plate again to identify the *E. coli* colonies presenting the blue color; 1 colony was selected for further analysis.

#### Antimicrobial susceptibility testing

The selected *E. coli* colony was subjected to antimicrobial susceptibility testing using the Vitek 2 automated system (Vitek AMS; bioMerieux Vitek Systems Inc., Hazelwood, MO, USA) with ID-GN and AST-N277 cards (Durham, NC, USA). The ASTN277 card was used to investigate ESBL production and antimicrobial susceptibility. The breakpoints of antimicrobial agents were determined according to Clinical and Laboratory Standards Institute standards [15]. Each panel had six wells containing cefepime (1.0 μg/mL), cefotaxime (0.5 μg/mL), and ceftazidime (0.5 μg/mL) alone and in combination with clavulanic acid (10, 4, and 4 μg/mL, respectively). The proportional reduction in growth in the wells containing cephalosporin + clavulanic acid compared with that in the wells containing cephalosporin alone was considered indicative of ESBL production. In this study, possible ESBL producers were screened using the M100-S19 (2009) breakpoints for the entire study period (Additional file 1).

MDR was defined as acquired nonsusceptibility to at least 1 agent in 3 or more antimicrobial categories, namely β-lactam or β-lactamase inhibitors (ampicillin and amoxicillin–clavunate), cephalosporins (flormoxef, cefazolin, cefuroxime, cefoxitin, cefotaxime, cefatazidime, and cefpirome), carbapenem (ertapenem and imipenem), aminoglycosides (gentamicin and amikacin), fluoroquinolones (ciprofloxacin and moxifloxacin), tigecycline, colistin, and TMP–SMZ [16].

#### Detection of O25b–ST131, multilocus sequence typing, and blaCTX-M gene groups

Polymerase chain reaction was performed as previously described [17] to screen for the O25b serotype by using the primers rfb.1bis (5′-ATACCGACGACGCCGATCTG-3′) and rfbO25b.r (5′-TGCTATTCATTATGCGCAGC-3′) [18, 19]. The sequence type was determined through multilocus sequence typing (MLST) by using the Achtman scheme (http://mlst.warwick.ac.uk/mlst/dbs/Ecoli) [20]. blaCTX-M groups 1, 2, and 9 were detected through multiplex polymerase chain reactions by using specific primers, as previously described [21]. Furthermore, specific polymerase chain reactions were performed to detect the common group 9 variant (CTX-M-14) and group 1 variant (CTX-M-15) [19, 20].

### Statistical analysis

All statistical analyses were performed using Stata Version 12.1 (StataCorp., College Station, Texas, USA) statistical software package. Categorical data were analyzed using the chi-squared and Fisher exact tests.

## Results

### Antimicrobial susceptibility testing

Stool samples were collected from 255 children; *E. coli* was cultured from 169 children. Twelve children were excluded because they were receiving antibiotic treatment or were hospitalized in the 3 months before specimen collection. Among 157 *E. coli* isolates, 25.5% (40/157) were susceptible to all tested antibiotics. Furthermore, 70.1% (110/157), 65.6% (103/157), 47.1% (74/157), and 32.5% (51/157) of the isolates were nonsusceptible to ampicillin, amoxicillin–clavunate, TMP–SMZ, and cefazolin, respectively. MDR *E. coli* accounted for 36.9% of all isolates (58/157; Table 1), among which 17.2% (27/157), 14.0% (22/157), and 5.7% (9/157) were nonsusceptible to at least 1 agent in 3, 4, and 5 antimicrobial categories, respectively.

**Table 1:** Antimicrobial nonsusceptibility rates of *E. coli* in the stools of community children in Southern Taiwan

Non-susceptible rate % (number/total number)				
	Total	MDR *E. coli* (*n* = 58)	Non MDR E. coli (*n* = 99)	p
Flormoxef	7.6 (12/157)	17.2 (10/58)	2.0 (2/99)	0.001
Ampicillin	70.1 (110/157)	100.0 (58/58)	52.5 (52/99)	< 0.001
Amoxicillin/clavunate	65.6 (103/157)	96.6 (56/58)	47.5 (47/99)	< 0.001
Cefazolin	32.5 (51/157)	77.6 (45/58)	6.1 (6/99)	< 0.001
Cefuroxime	22.9 (36/157)	55.2 (32/58)	4.0 (4/99)	< 0.001
Cefuroxime Axetil	24.8 (39/157)	55.2 (32/58)	7.1 (7/99)	< 0.001
Cefoxitin	15.3 (24/157)	37.9 (22/58)	2.0 (2/99)	< 0.001
Cefotaxime	19.6 (31/157)	46.6 (27/58)	4.0 (4/99)	< 0.001
Ceftazidime	13.4 (21/157)	29.3 (17/58)	4.0 (4/99)	< 0.001
Cefpirome	8.3 (13/157)	19.0 (11/58)	2.0 (2/99)	< 0.001
Ertapenem	0.0 (0/157)	0.0 (0/58)	0.0 (0/99)	NA
Imipenem	0.0 (0/157)	0.0 (0/58)	0.0 (0/99)	NA
Amikin	0.0 (0/157)	0.0 (0/58)	0.0 (0/99)	NA
Gentamicin	22.9 (36/157)	60.3 (35/58)	1.0 (1/99)	< 0.001
Ciprofloxacin	18.5 (29/157)	43.1 (25/58)	4.0 (4/99)	< 0.001
Moxifloxacin	19.1 (30/157)	43.1 (25/58)	5.1 (5/99)	< 0.001
Tigecycline	0.0 (0/157)	0.0 (0/58)	0.0 (0/99)	NA
Colistin	0.6 (1/157)	1.7 (1/58)	0.0 (0/99)	0.190
Trimethoprim-sulfamethoxazole	47.1 (74/157)	84.5 (49/58)	25.3 (25/99)	< 0.001

*NA* non-appreciable

Thirteen *E. coli* isolates produced ESBL; 11 were MDR *E. coli*. Twenty-nine *E. coli* isolates were nonsusceptible to ciprofloxacin; 25 were MDR *E. coli* (Table 2).

**Table 2:** Number of *E. coli* isolates with/without ESBL, susceptible/nonsusceptible ciprofloxacin, O25b, and ST131 in children’s stool

	ST 131		Non ST 131	
	O25b	NonO25b	O25b	NonO25b
ESBL + (*n* = 13)	7	4	2	0
ESBL – (*n* = 144)	3	3	14	124
Cipro R (*n* = 29)	6	1	4	18
Cipro S (*n* = 128)	4	2	12	110

### MLST of *E. coli* isolates

Twenty-six *E. coli* isolates were O25b positive, among which 9 were MDR *E. coli* and 9 were ESBL producers. Among 58 MDR *E. coli* isolates, 7 were O25b–ST131 positive and ESBL producers. Only 1 isolate was positive for O25b–ST131 but was not MDR and did not produce ESBL. Seven isolates with O25b–ST131 positivity were MDR *E. coli*, among which only 1 was susceptible to ciprofloxacin. Furthermore, 1 isolate with O25b–ST131 positivity was not MDR and was susceptible to ciprofloxacin. Compared with the non-ESBL and ciprofloxacin-susceptible groups, the ESBL and ciprofloxacin-nonsusceptible groups had significantly higher rates of O25b–ST131 positivity (4.2% vs 53.9 and 4.70% vs 24.1%, respectively; Table 3).

**Table 3:** Rates of *E. coli* ST131 positivity of the ESBL, non-ESBL, ciprofloxacin-nonsusceptible, ciprofloxacin-susceptible, MDR, and non-MDR groups

	ST131 positive % (number/total number)
ESBL n = 13	53.6% (7/13)
Non-ESBL n = 144	4.2% (6/144)
P	**<** 0.001
Ciprofloxacin non-susceptible (n = 29)	24.1% (7/29)
Ciprofloxacin susceptible (n = 128)	4.7% (6/128)
P	0.003
MDR (n = 58)	13.8% (8/58)
Non-MDR (n = 99)	5.1% (5/99)
P	0.073

### blaCTX-M gene study

Five of the 13 (38.5%) ESBL-producing *E. coli* belonged to blaCTX-M group 9, among which were all CTXM-14, and 4 (80%) were confirmed as ST131. Three O25b ESBL-producing *E. coli* contained the CTX-M group 1 genes that were confirmed to be CTX-M-(3, 15), and they were all ST131 (Table 4).

**Table 4:** Number of CTX-M genes detected in O25b and non-O25b ESBL-producing *E. coli* and ESBL-nonproducing *E. coli*

*E. coli*	Total number: 157																			
	ESBL										Non-ESBL									
	13										144									
Type	O					NO					O					NO				
No.	9					4					17					127				
ST	69	73	95	131	NS	69	73	95	131	NS	69	73	95	131	NS	69	73	95	131	NS
No.	0	0	0	7	2	0	0	0	0	4	1	0	0	3	13	9	5	5	3	105
CTX-M-G1	0	0	0	0	0	0	0	0	0	0	0	0	0	0	0	0	0	0	1	1
CTX-M-G1 CTX-M-(3,15)	0	0	0	3	0	0	0	0	0	0	0	0	0	0	0	0	0	0	0	1
CTX-M-G2	0	0	0	0	0	0	0	0	0	0	0	0	0	0	0	0	0	0	0	0
CTX-M-G8	0	0	0	0	0	0	0	0	0	1	0	0	0	0	0	0	0	0	0	0
CTX-M-G9 CTX-M-14	0	0	0	4	0	0	0	0	0	1	1	0	0	1	2	2	0	0	0	5
Non-CTX-M-group-(1,2,8,9)	0	0	0	0	2	0	0	0	0	2	0	0	0	2	11	7	5	5	2	98

O, O25b; NO, Non O25b; NS, Non ST.

## Discussion

Antimicrobial resistance in commensal flora is a serious threat because a very highly populated ecosystem, such as the gut, may become a source of additional intestinal infections at a later stage. These infections may subsequently spread to other hosts or transfer genetic resistance elements to other members of the microbiota, including pathogens [22]. A major public health concern is that with the increasing number of individuals carrying these resistant strains as part of their normal flora, the probability of acquiring clinical infections, either in the community or hospital, increases. Several studies have addressed the prevalence of resistant *E. coli* isolated from the stools of children [23–38] (Table 5). The rates of resistance to first-line antimicrobial agents, namely ampicillin, TMP–SMZ, and first-generation cephalosporins, were 16–100%, 8–100%, and 6–10%, respectively. Furthermore, the rate of resistance to ciprofloxacin was 0–53%, and 2.6–20.3% of *E. coli* were ESBL producers. The aforementioned studies had varied methodologies, study periods, sample sizes, and demographics and should thus be compared cautiously. However, the importance of surveillance of resistant strains must be addressed to achieve a holistic strategy for resistance control.

**Table 5:** Summary of 20 studies on the fecal carriage of *E. coli* in community children from different countries

Country (published years)	First author	Study population	Setting	Sample size	Prevalence of resistance
Houston, Tex USA (1987) [23]	Reves RR	Children	Cross-section, day-care centers	79	trimethoprim: 37%; ampicillin: 70%
Bolivia (1998) [24]	Bartoloni A	aged 6–72 months	healthy children; community-based	296	Ampicillin: 97% TMP/SMX: 94%, Cephalothin:10% Tetracycline: 92% Ciprofloxacin: 0%
Shanghai, China (1998) [25]	Zhang XL	Group A: Children of 5–6 years Group B: Children of 10–11 years	A: Nursery school B: Primary school	A: 30 B: 54	A: Ampicillin: 93.3% Trimethoprim: 100% Ciprofloxacin: 43.3% B: Ampicillin: 100% Trimethoprim: 100% Ciprofloxacin: 53.7%
Mexican (2003) [26]	Zaidi MB	healthy children (1 month to 12 years)	day care centers or kindergartens	276	nalidixic acid: 54% ciprofloxacin: 18.5%
Bolivia and Peru (2006) [27]	Bartoloni A	children (aged 6 to 72 months)	Health children in four urban area	3174	Ampicillin: 95%, trimethoprim-sulfamethoxazole: 94% nalidixic acid: 35% gentamicin: 21% ciprofloxacin: 18% ceftriaxone: 0.1% amikacin: 0.1%
Germany (2007) [28]	Lietzau S	children aged 6 months to 4 years	regular health screening or an acute infection	884	Ampicillin: 16.6% amoxicillineclavulanic acid: 8% cotrimoxazole: 8.7% Nalidixic acid: 2.0% Levofloxacin: 0.4%
India (2009) [29]	Seidman JC	aged 5–10 years	primary school children	119	Cefazoline: 6.7% (8/119) ampicillin: 38.7% (46/119) cotrimoxazole: 37.0% (44/119) Ciprofloxacin: 12.6% (15/119) Cefotaxime: 4.2% (5/119)
Senegal (2009) [30]	Ruppé E	aged 1 to 11 years	healthy children	20	ESBL-producing: 10%
Portugal (2009) [31]	Guimaraes B	Aged 1 to 14 years	healthy children	112	ESBL-positive: 2.7%
Vietnam (2012) [32]	Dyar OJ	child aged 6–60 months	rural children (1% with diarrhea)	818	Ampicillin: 65% co-trimoxazole: 68% ciprofloxacin: < 1%
Guinea-Bissau (2012) [33]	Isendahl J	children < 5 years of age	fever or tachycardia attending a pediatric emergency ward	408	ESBL-producing E coli: 20.34% (83/408)
Sweden (2013) [34]	Kaarme J	Children (range 11–66 months) 66 months	preschool	313	ESBL-producing E coli: 2.6% (8/313)
Libyan (2014) [35]	Ahmed SF	children aged from 3 to 12 years	Diarrhea attending outpatient clinics	134	Ampicillin: 78.4% Amoxicillin/Clavulanic: 64.2% TMP/SMZ: 61.9% Cefotaxime: 20.2% Ciprofloxacin: 5.2% ESBL-producing E coli: 13.4% (18/134)
France (2014) [36]	Blanc V	Children (3 and 40 months)	Day-care center	419	ESBL-producing E coli: 6.4%
Spain (2014) [37]	Fernández-Reyes M	children at the ages of 8, 12, and 16 months	healthy children in the community	125	ESBL-producing E coli: 24% of 125 children and 10.7% of the 318 fecal samples
Lao People’s Democratic Republic (2015) [38]	Stoesser N	children ≤6 years of age	preschool childcare facilities	397	ESBL-producing E coli: 19.65% (78/397) Amoxicillin/clavulanate: 10.33% (41/397) Co-trimoxazole: 14.61% (58/397) Cefotaxime: 19.4% (77/397) Ofloxacin: 3.78% (15/397)

The present study is the first to investigate the fecal carriage of MDR *E. coli* in community children in Taiwan. In this study, high rates of nonsusceptibility to commonly used antimicrobial agents, such as ampicillin, amoxicillin + clavulanate, TMP–SMZ, and cefazolin (70, 65.6, 47.1, and 32.5%, respectively) were obtained. The rate of nonsusceptibility to ciprofloxacin was approximately 18.3%. By contrast, the rate of nonsusceptibility to relatively rarely used antibiotics, which could only be prescribed by infection doctors in medical centers in Taiwan, such as imipenem, amikacin, tigecycline, and colistin, was 0%. This phenomenon could be explained by the selective pressure induced by the intensive use of antibiotics commonly used in both humans and nonhumans. Therefore, under the pressure of excessive antibiotic use, genes, such as blaCTX-M, spread amongst different bacterial species and strains through horizontal gene transfer and thus contribute to the rapid dispersal of antibiotic resistance in the community [39]. In our study, MDR *E. coli* accounted for 36.9% of all isolates. Although this percentage is lower than that reported in Guinea-Bissau [33], the rates of nonsusceptibility to amoxicillin + clavulanate and ciprofloxacin and of ESBL-producing *E. coli* are still higher than those reported in developed countries [28, 36].

In our previous study of 111 infants who were hospitalized for UTIs caused by ESBL-producing *E. coli,* O25b–ST131 was identified in 65% of isolates [40]. Among the 111 patients, 92 isolates belonged to blaCTX-M group 9, and most were CTXM-14. Furthermore, most patients with bacteremia or UTIs were previously healthy and did not exhibit any apparent risk factors, including previous antimicrobial use, hospitalization, neonatal infection, and underlying disease. Only 30% of the infants with UTIs caused by this clone had identifiable risk factors. Our findings support that most drug-resistant *E. coli* infections are community—not hospital—acquired [17, 40, 41]. Furthermore, we previously evaluated *E. coli* isolated from several rivers in Southern Taiwan and revealed that the most commonly isolated ESBL-producing *E. coli* clonal complexes were ST10 and ST58 and not the major clone ESBL-producing *E. coli* ST131, which causes community-acquired infections both worldwide and in Taiwan [42]. In contrast, in the present study, ST131 (7/13) was the most common ESBL-producing *E. coli* clone in the stools of children.

In the present study, 13 *E. coli* isolates produced ESBL, of which 11 were MDR *E. coli* and 69% (9/13) were nonsusceptible to ciprofloxacin. These results corroborate the finding that the plasmid-mediated transfer of ESBLs enhances resistance to non-β-lactams, such as quinolones, because plasmids can harbor genes that confer resistance to multiple antibiotic groups [43, 44]. Furthermore, compared with the non-ESBL and ciprofloxacin-susceptible groups, the ESBL and ciprofloxacin-nonsusceptible groups yielded significantly higher rates of ST131 positivity. The rates of ST131 positivity tended to be higher in the MDR group than in the non-MDR group (13.8% vs 5.1%, *p* = 0.073). These results also demonstrated that the *E. coli* ST131 clone might be associated with antimicrobial resistance.

Our study has some limitations. First, we used a hospital-based design; therefore, our findings are not generalizable to healthy children in the community because of potential selection bias in patient inclusion. Furthermore, the fecal samples in this study were collected within 3 days of hospitalization. Transmission leading to asymptomatic carriage may occur within less than 3 days after hospital admission. Second, to prevent risk factors from affecting the fecal carriage of resistant *E. coli*, patients who received antibiotic treatment or were hospitalized in the 3 months before specimen collection as well as those with underlying diseases were excluded, leading to the possible underestimation of antimicrobial resistance. Third, because the diseases of the enrolled patients were not further classified, we could not determine whether the different diseases affected the fecal carriage and antimicrobial resistance rate of *E. coli*. For example, including children with gastroenteritis may cause bias because the infection may lead to a temporary change in intestinal flora, with a dominance of pathogenic bacteria. In addition, identifying *E. coli* based on the blue color of the CHROMagar™ ECC may cause misidentification because other species, such as *Enterobacter*, *Klebisella*, and *Citrobacter*, also showed a blue color, which may overestimate the prevalence of *E. coli*. However, selecting one isolate per sample for testing may have underestimated the prevalence of *E. coli* in this study.

## Conclusion

This study is the first to report a high rate (37%) of MDR *E. coli* in the stools of community children in Southern Taiwan. Twenty-nine *E. coli* isolates (18.5%) were not susceptible to ciprofloxacin, and approximately 8.3% of *E. coli* produced ESBL. O25b–ST131 (7/13) was the most common ESBL-producing *E. coli* clonal group in the stools of children. These results highlight the importance of establishing an antibiotic stewardship and infection control programs to reduce inappropriate antibiotic use and limit the transmission of MDR *E. coli*.

## Additional file

Additional file 1: **Table S1.** Minimal inhibitory concentration breakpoints for Enterobacteriaceae. (DOCX 16 kb)

## Abbreviations

*E. coli*: *Escherichia coli*
ESBL: Extended-spectrum β-lactamase
MDR: Multidrug-resistant
MLST: Multilocus sequence typing
TMP–SMZ: Trimethoprim–sulfamethoxazole
UTIs: Urinary tract infections
