# Supplementary material for: Fecal carriage of multidrug-resistant Escherichia coli by community children in southern Taiwan
Source: BMC Gastroenterol. 2018 Jun 15;18:86. doi: 10.1186/s12876-018-0807-x (PMC6003077; doi:10.1186/s12876-018-0807-x)
Supplement: Supplementary file 1 — Table S1. Minimal inhibitory concentration breakpoints for Enterobacteriaceae. (DOCX 16 kb) [file 12876_2018_807_MOESM1_ESM.docx]

Table S1 Minimal inhibitory concentration breakpoints for Enterobacteriaceae

| Antimicrobial  agents | Interpretive categories and MIC^@^ breakpoints (µg/ml) | | |
| --- | --- | --- | --- |
|  | S | I | R |
| Flormoxef | ≦8 | 16-32 | ≧64 |
| Ampicillin | ≦8 | 16 | ≧32 |
| Amoxicillin/clavunate | ≦8/4 | 16/8 | ≧32/16 |
| Cefazolin | ≦16 | - | ≧32 |
| Cefuroxime | ≦8 | 16 | ≧32 |
| Cefuroxime Axetil | ≦4 | 8-16 | ≧32 |
| Cefoxitin | ≦8 | 16 | ≧32 |
| Cefotaxime | ≦1 | 2 | ≧4 |
| Cefatazidime | ≦4 | 8 | ≧16 |
| Cefpirome | ≦ |  | ≧ |
| Ertapenem | ≦0.5 | 1 | ≧2 |
| Imipenem | ≦1 | 2 | ≧4 |
| Amikin | ≦16 | 32 | ≧64 |
| Gentamicin | ≦4 | 8 | ≧16 |
| Ciprofloxacin | ≦1 | 2 | ≧4 |
| Moxifloxacin | ≦2 | 4 | ≧8 |
| Tigecycline | ≦2 | 4 | ≧8 |
| Colistin | ≦2 | - | >2 |
| Trimethoprim -sulfamethoxazole | ≦2/38 | - | ≧4/76 |

^@^ MIC: minimal inhibitory concentration
